# Interpreting the efficacy enhancement mechanism of Chinese medicine processing from a biopharmaceutic perspective

**DOI:** 10.1186/s13020-024-00887-0

**Published:** 2024-01-18

**Authors:** Bing Yang, Zhubin Zhang, Jinjing Song, Tianhao Qi, Jingqi Zeng, Liang Feng, Xiaobin Jia

**Affiliations:** State Key Laboratory of Natural Medicines, School of Traditional Chinese Pharmacy, China Pharmaceutical University, Nanjing, 211198 People’s Republic of China

**Keywords:** Chinese medicine processing, Biopharmaceutical properties, Processing adjuvants, Phase transition, Absorption, Chemical reaction

## Abstract

Chinese medicine processing (CMP) is a unique pharmaceutical technology that distinguishes it from natural medicines. Current research primarily focuses on changes in chemical components to understand the mechanisms behind efficacy enhancement in processing. However, this paper presents a novel perspective on the biopharmaceutics of CMP. It provides a comprehensive overview of the current research, emphasizing two crucial aspects: the role of ‘heat’ during processing and the utilization of processing adjuvants. The paper highlights the generation of easily absorbed components through the hydrolysis of glycosides by ‘heat’, as well as the facilitation of dissolution, absorption, and targeted distribution of active components through the utilization of processing adjuvants. From a biopharmaceutic perspective, this paper provides a lucid comprehension of the scientific foundation for augmenting the efficacy of CMP. Moreover, it proposes a three-dimensional research framework encompassing chemical reactions, phase transitions, and biopharmaceutical properties to further investigate the mechanisms involved in enhancing the efficacy of CMP.

**Graphical Abstract:**

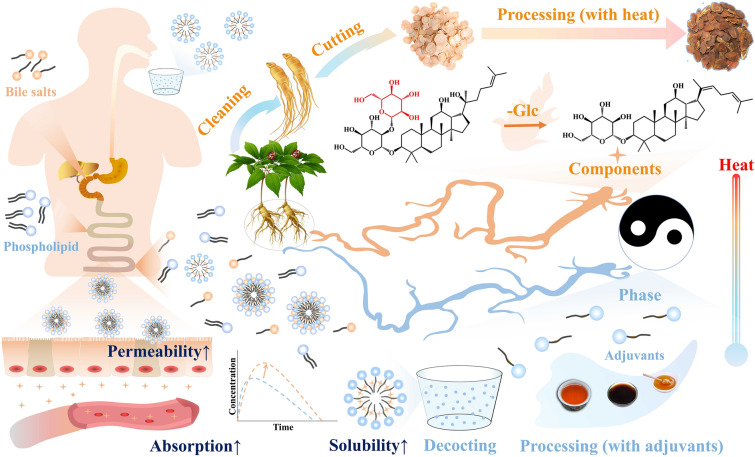

## Introduction

Chinese medicinal materials cannot be used directly in clinical practice but must undergo processing according to Chinese medicine processing theory to become decoction pieces that are suitable for use in clinical prescriptions. Processing is a unique pharmaceutical technology in China and involves various methods, such as stir-frying, steaming, and boiling [1–4]. Furthermore, common adjuvants like vinegar, honey, wine, salt solution, ginger juice, bran, and rice are frequently employed during processing [5–8]. Over millennia of clinical practice, the scope of clinical application for Chinese medicine has expanded, producing various decoction pieces that undergo processing with adjuvants [9, 10]. The Chinese Pharmacopeia (2020 edition) lists decoction pieces and their related processing methods as specific items of Chinese medicines.

While the classical processing theory has proven reliable in long-term clinical practice, the scientific principles underlying it are not fully understood to date. Gaining a thorough understanding of the chemical reactions and changes in chemical components during processing is crucial for uncovering alterations in clinical efficacy before and after CMP [11, 12]. Currently, the explanation of the CMP mechanism mainly focuses on the changes in chemical components. Although recent research has shed light on some of these changes, the modifications in components often do not align with pharmacological effects. In other words, the changes in component content following processing cannot fully elucidate the scientific significance of the CMP. This suggests that the efficacy of Chinese medicine is not only influenced by changical components but is also closely related to biopharmaceutical processes within the body.

The low bioavailability of active components in Chinese medicine is a critical factor that affects its effectiveness. This is closely associated with the biopharmaceutical properties of these active components, such as poor solubility, low dissolution rate, low mucosal permeability, and limited absorption in the gastrointestinal tract [13–15]. The processes of absorption, distribution, metabolism, and excretion of active components in the gastrointestinal tract significantly influence their biological effects, with absorption playing a vital role in determining efficacy [16, 17]. The poor solubility and low permeability of most active components in Chinese medicine hinder their absorption in the gastrointestinal tract [18–20]. Furthermore, studies have demonstrated that the combination of adjuvants and active components can form unique morphologies, which can alter the solubility, permeability, and intestinal absorption of the active components, ultimately enhancing their therapeutic effectiveness. Therefore, to comprehensively understand the mechanisms of CMP, it is crucial to not only investigate the changes in chemical components during processing in vitro but also gain insights into the changes that occur in vivo. This involves elucidating the solubility, permeability, gastrointestinal absorption, and tissue distribution of active components.

Based on the overview of chemical reactions and changes in active components during processing, our research team has proposed an innovative approach to investigating the mechanism of CMP from a biopharmaceutical perspective. Our emphasis is on improving the biopharmaceutical properties of active components under the dual influence of heat and adjuvants. In addition to the hydrolysis reaction that occurs under the heating effect, resulting in the formation of easily absorbed components, we propose that the special formulation characteristics of adjuvants can establish a specific form with active components. This alteration in form can subsequently modify the solubility and permeability of the active components, thereby enhancing their in vivo biopharmaceutical behavior. This paper provides a clearer understanding of the scientific basis for enhancing the efficacy of CMP from a biopharmaceutic perspective, providing a novel perspective for revealing the scientific significance of CMP.

## Formation of absorbable components during processing

For active components to be effective, they must be absorbed into the bloodstream (except for direct intestinal action and external application). Although Chinese medicine contains numerous components, it is commonly believed that only those components that are absorbed into the bloodstream can exert their effects [21, 22]. When evaluating the CMP from a biopharmaceutic perspective, it becomes apparent that the processing transforms these components, thereby enhancing their biopharmaceutical properties. This transformation enhances the absorption of these components into the bloodstream, resulting in an increased content of active components in the body. This could be a significant contributing factor to the observed improvement in efficacy during processing.

### Hydrolysis of glycoside to produce easily absorbed components by “heat”

Hydrolysis is a frequently occurring chemical reaction during processing. Active components, such as flavonoid glycosides, saponins, iridoid glycosides, and polysaccharides, undergo hydrolysis reactions. These reactions can effectively reduce the number of glycosyl groups present in these components, consequently increasing their permeability in the body.

#### Flavonoid glycosides

**Fig. 1:**
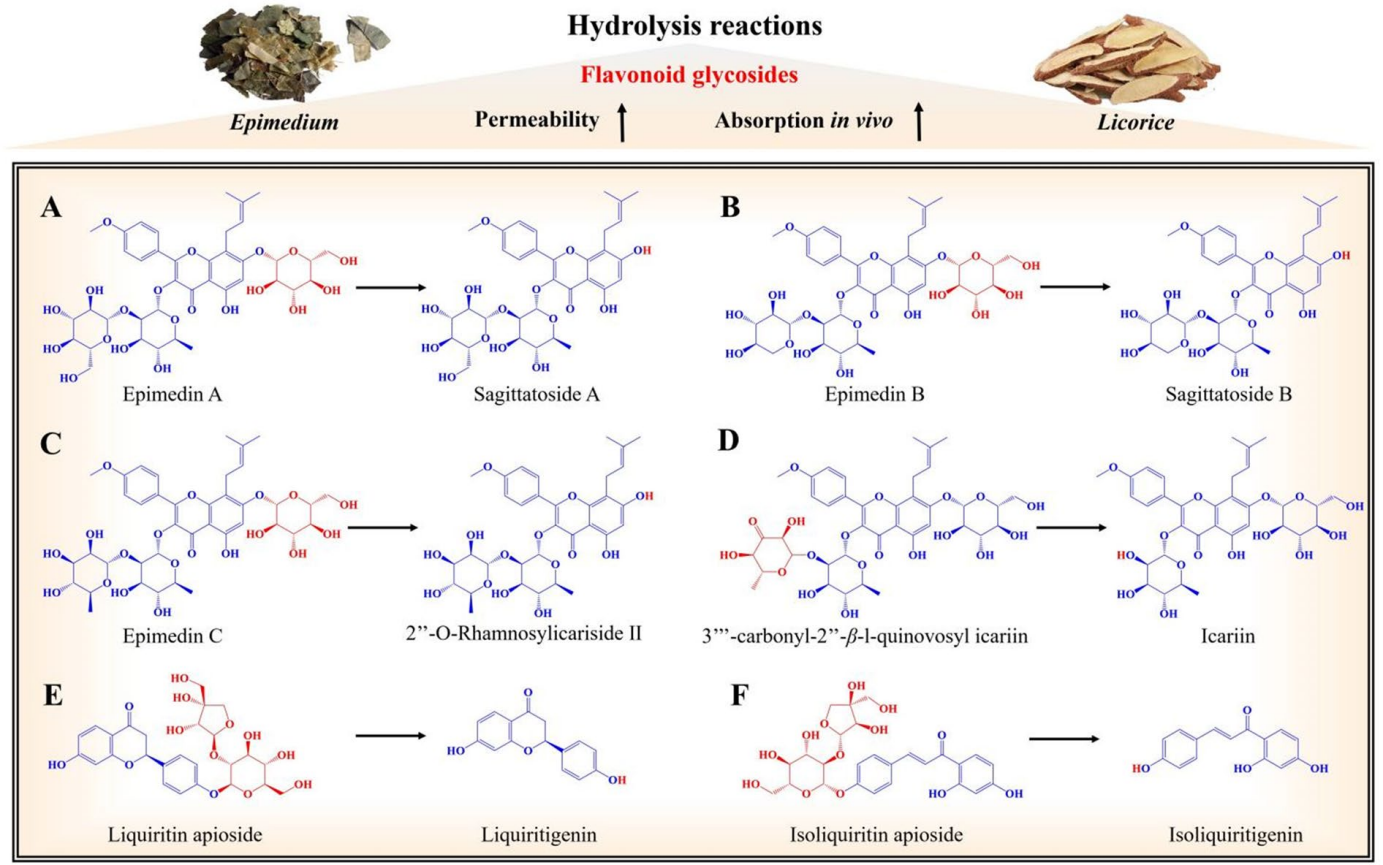
Hydrolysis reaction of flavonoid glycoside components during processing. **A** Epimedin A is hydrolyzed into sagittatoside A; **B** Epimedin B is hydrolyzed into sagittatoside B; **C** Epimedin C is hydrolyzed into 2″*-O-*rhamnosylicariside II; **D** 3‴-carbonyl-2″-*β*-l-quinovosyl icariin is hydrolyzed into icariin; **E** liquiritin apioside is hydrolyzed into liquiritigenin; **F** isoliquiritin apioside is hydrolyzed into isoliquiritigenin

Flavonoid glycosides are widely present in various types of Chinese medicine, such as Epimedh Folium (Yinyanghuo in Chinese), and Glycyrrhizae Radix et Rhizoma (Gancao in Chinese). Epimedh Folium, contains three-glycosyl flavonoid glycosides (epimedin A, epimedin B, epimedin C, 3‴-carbonyl-2″-*β*-l-quinovosyl icariin), two-glycosyl flavonoid glycosides (sagittatoside A, sagittatoside B, 2-*O-*Rhamnosylicariside II, Icariin) and one-glycosyl flavonoid glycosides (baohuoside I), which are considered the primary active components. Research has shown that one- or two-glycosyl flavonoid glycosides are more easily absorbed than those with three-glycosyl, as they not only ensure suitable solubility but also exhibit better permeability in the body [23, 24]. When Epimedh Folium is stir-fried with mutton tallow, hydrolysis reactions occur, converting epimedin A to sagittatoside A, epimedin B to sagittatoside B, epimedin C to 2″*-O-*Rhamnosylicariside II, and 3‴-carbonyl-2″-*β*-l-quinovosyl icariin to icariin (Fig. 1A–D) [25–27]. This indicates that the heat employed during processing can reduce the number of glycosyl groups on flavonoid glycosides in Epimedh Folium through hydrolysis reactions. Consequently, this process enhances their permeability, promotes absorption, increases their concentration in the body, and overall enhances the effectiveness of Epimedh Folium.

Liquiritin apioside and isoliquiritin apioside are flavonoid glycosides commonly found in Glycyrrhizae Radix et Rhizoma. However, for them to be absorbed into the bloodstream, they require the assistance of digestive tract microorganisms to convert them into liquiritigenin and isoliquiritigenin, respectively [28]. Alternatively, when Glycyrrhizae Radix et Rhizoma is stir-fried under heat, liquiritin apioside and isoliquiritin apioside into liquiritigenin and isoliquiritigenin (Fig. 1E, F) [29, 30]. This process facilitates the absorption of active flavonoids into the bloodstream, leading to an increase in their concentration in the body. Therefore, processing plays a crucial role in enhancing the effectiveness of Glycyrrhizae Radix et Rhizoma after processing.

#### Saponins

Saponins can be classified into two categories based on their aglycone structures: triterpenoid saponins and steroidal saponins. These components are abundant in Chinese medicine, with Glycyrrhizae Radix et Rhizoma, Rhizoma Anemarrhenae (Zhimu in Chinese), and Ginseng Radix et Rhizoma (Renshen in Chinese) being rich sources of saponins. During processing with heat, saponins may undergo hydrolysis, leading to the removal of their glycosyl groups.

**Fig. 2:**
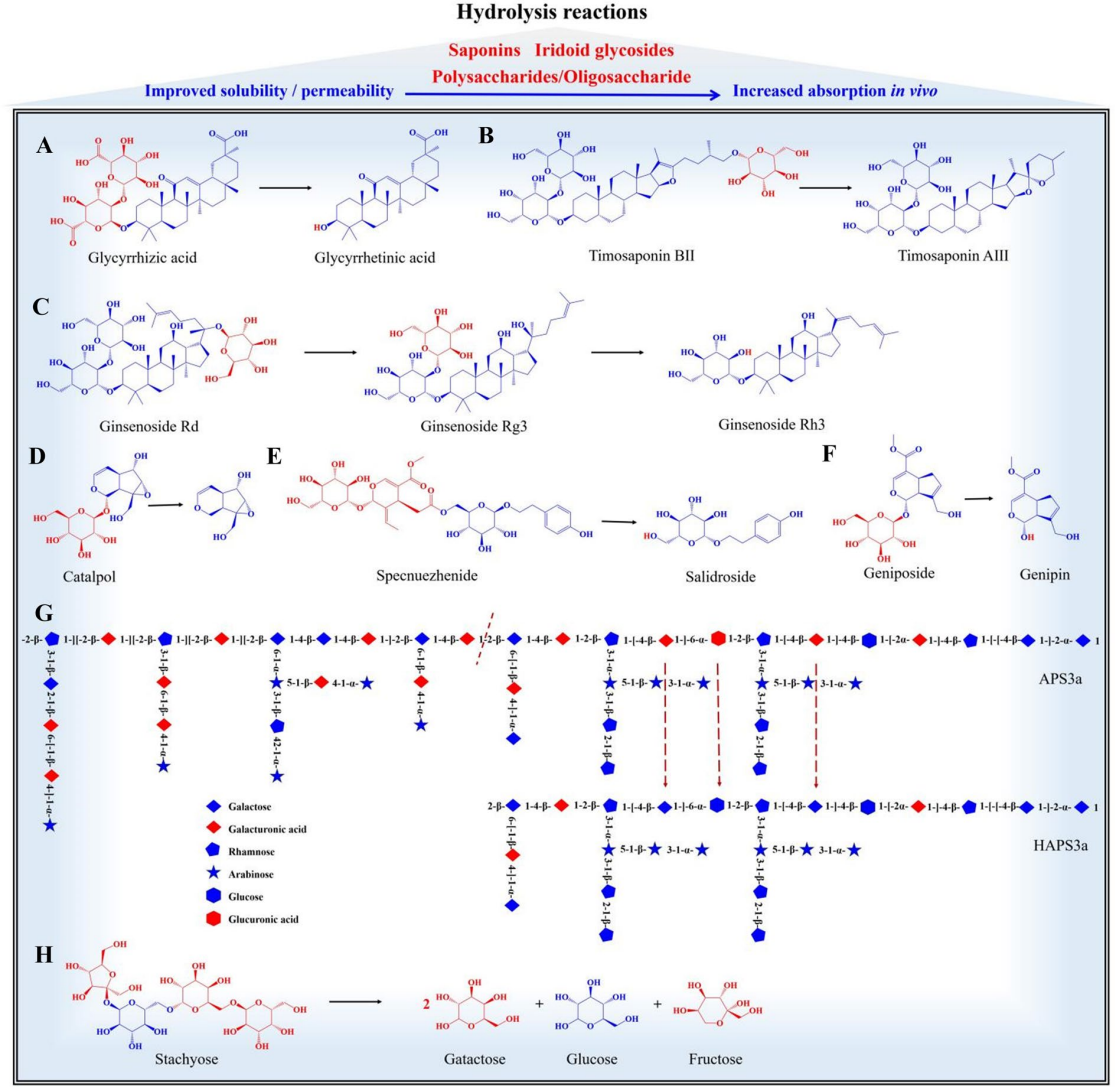
Hydrolysis reaction of saponins, iridoid glycosides, polysaccharides/oligosaccharide during processing. **A** Glycyrrhizic acid is hydrolyzed into glycyrrhetinic acid; **B** timosaponin BII is hydrolyzed into timosaponin AIII; **C** ginsenoside Rd undergoes deglycosylation at the C20 position to yield ginsenoside Rg3, which further hydrolyzes into generate Rh3; **D** monoglycosides iridoid glycosides catalpol is hydrolysed; **E** specnuezhenide is hydrolyzed into salidroside; **F** geniposide is hydrolyzed into genipin; **G** APS3a undergoes a hydrolysis reaction, converting the 1,4-β-Galpa and 1,6-α-GlcpA aldehyde acid residues into HAPS3a; **H** stachyose is hydrolyzed into two molecules of galactose, one molecule of fructose, and one molecule of glucose

Glycyrrhizae Radix et Rhizoma is known for its ability to invigorate the spleen and *Qi*, relieve urgency and pain, and harmonize with other medicines. Its primary component, glycyrrhizic acid, is a typical triterpenoid saponin [31]. However, studies have indicated that the oral bioavailability of glycyrrhizic acid is low, at only 4.0%. It is mainly absorbed through direct or stepwise removal of two molecules of glucuronic acid by intestinal microbial hydrolysis, which generates glycyrrhetinic acid. Glycyrrhetinic acid has a high bioavailability of up to 90% and is the primary form of glycyrrhizic acid that is absorbed into the bloodstream and exerts its effects [32]. Several factors influence the transport of active components across membranes. These factors include the expression and activity of efflux transporters, the strength of membrane permeability, and the state of tight junctions between cells. When triterpenoid saponins undergo deglycosylation and turn into sapogenins, their polarity decreases while their fat solubility increases. As a result, they become more easily absorbed in the intestine. In vivo, glycyrrhetinic acid demonstrates excellent absorption properties. It can further enhance its absorption in the gastrointestinal tract by inhibiting efflux transporters such as P-gp, MRP, and BCRP [33, 34]. During the stir-frying of Glycyrrhizae Radix et Rhizoma, glycyrrhizic acid undergoes hydrolysis due to heat, resulting in the formation of the easily absorbed active component glycyrrhetinic acid (Fig. 2A) [35].

Rhizoma Anemarrhenae possesses medicinal properties such as heat-clearing, *fire-*purging, *yin-*nourishing, and dryness-moistening. Its steroidal saponins are considered the main active components, which can be classified into two categories based on the aglycone structure: spirostanol saponins and furostanol saponins. Among these, Timosaponin BII is the most abundant component, accounting for over 70% of the total steroidal saponins in Rhizoma Anemarrhenae [36]. However, the absolute bioavailability of Timosaponin BII in rats was found to be only 0.26%. Nevertheless, research has indicated that processing Rhizoma Anemarrhenae (by str-frying with salt solution) can hydrolyze timosaponin BII into timosaponin AIII under the influence of heat, as illustrated in Fig. 2B [37]. This suggests that processing can enhance the efficacy of Rhizoma Anemarrhenae by promoting the transformation of steroidal saponins into easily absorbable components, such as timosaponin AIII, timosaponin AI, and sarsasapogenin. These are the primary forms that are absorbed into the bloodstream [38, 39].

Ginsenosides, the primary active components of Ginseng Radix et Rhizoma, mainly consist of dammarane-type ginsenosides. These include ginsenosides Rb1, Rc, Rb2, Rd, Re, and Rg1, which are the most abundant [32, 40]. However, due to their high relative molecular mass and structural characteristics, they have poor membrane permeability, low water solubility, and are difficult to absorb in the gastrointestinal tract. Consequently, the oral bioavailability of almost all ginsenosides is low. For instance, the oral bioavailability of ginsenosides Rb1 and Rb2 is only 0.78% and 0.08%, respectively [41]. The presence of glycosyl groups is the main factor contributing to the differences in absorption among various ginsenosides. The higher the number of glycosyl groups, the more hydrogen bonds are formed, resulting in reduced membrane permeability and decreased absorption of most ginsenosides. Therefore, ginsenosides with multiple glycosides are challenging to absorb in vivo [42].

Numerous studies have demonstrated that ginsenosides are not hydrolyzed in the stomach when orally administered. Only a small portion of ginsenosides are absorbed in their original form directly through the small intestine. The primary forms of ginsenosides that are absorbed into the bloodstream and exhibit their medicinal effects are the rare saponins (Rg3, Rg5, Rh2, Rh3, Rk1, Rk2), and the glycosides produced after hydrolysis [43–45]. During the steaming process of Ginseng Radix et Rhizoma, ginsenosides containing more glycosyl groups can undergo hydrolysis, resulting in the removal of glycosyl groups and the formation of ginsenosides with fewer glycosyl groups. Specifically, during steaming, the protopanaxatriol-type saponin ginsenoside Rd can undergo deglycosylation at the C20, producing ginsenoside Rg3. Furthermore, ginsenoside Rg3 can undergo further deglycosylation at the C3 position, leading to the production of ginsenoside Rh2 [46–48]. Studies have shown that newly formed ginsenosides in the form of mono-glycoside or aglycone are more easily absorbed in the gastrointestinal tract compared to primary saponins that contain more glycosyl groups, such as Ginsenoside Rd [49].

#### Iridoid glycosides

Iridoids can be categorized into two primary chemical structures: cyclopentane iridoids and cyclopentane-cracked secoiridoids. They are predominantly present as iridoid glycosides and are commonly found in Chinese medicine derived from the Scrophulariaceae, Rubiaceae, and Oleaceae families, such as Rehmanniae Radix (Dihuang in Chinese), Gardeniae Fructus (Zhizi in Chinese) and Ligustri Lucidi Fructus (Nvzhenzi in Chinese). However, iridoid glycosides are susceptible to hydrolysis and thus exhibit instability. During processing, the glycosidic bonds of iridoid glycosides can be cleaved under the influence of heat, resulting in the loss of glycosyl groups through hydrolysis. For example, during the steaming process of Rehmanniae Radix into Rehmanniae Radix Praeparata (Shudihuang in Chinese), iridoid glycosides undergo various degrees of hydrolysis reactions [50]. The extent of hydrolysis is correlated with the number of glycosyl groups, with monoglycosides iridoid glycosides like catalpol being the most prone to hydrolysation (Fig. 2D).

Ligustri Lucidi Fructus is composed mainly of cyclopentane-cracked secoiridoid glycosides [51, 52]. These glycosides are structurally linked to salidroside, tyrosol, or hydroxytyrosol through an ester bond on the cracked cyclopentane. For instance, specnuezhenide and ligustroside G13 are connected to salidroside, while oleuropein and ligustroside are associated with hydroxytyrosol and tyrosol via the ester bond. During processing (steaming), hydrolysis reactions can occur in the iridoid glycosides. This leads to a decrease in the levels of specnuezhenide, ligustroside G13, oleuropein, and ligustroside, while the levels of salidroside, tyrosol, and hydroxytyrosol increase. It has been observed that specnuezhenide can be hydrolyzed to salidroside under heat (Fig. 2E) [53, 54]. The wine steaming of Ligustri Lucidi Fructus enhances its renal protective function by hydrolyzing its iridoid glycosides [55]. This hydrolysis causes changes in the components from large to small molecular weight, facilitating their absorption in the intestinal tract [56].

Gardeniae Fructus contains geniposide, a cyclopentane iridoid glycoside known for its beneficial effects. Although geniposide’s bioavailability is limited due to its poor lipid solubility, despite being highly soluble in water. In rats, oral administration of geniposide resulted in only 4.23% absolute bioavailability compared to intravenous administration. The absorption of geniposide is rapid, occurring within 30 min [57]. When orally administered, most geniposide undergoes deglycosylation by intestinal bacteria and is absorbed into the bloodstream as genipin, its aglycone form [58]. It is commonly believed that the prototype components contained in Chinese medicine undergo metabolism and transformation in vivo, leading to enhanced effects. Processing techniques can facilitate the conversion of difficult-to-absorb components into easily absorbed active components. For example, during processing, geniposide can be hydrolyzed to genipin through heat (Fig. 2F) [59], which increases the content of active components in the body.

#### Polysaccharides/oligosaccharide

Polysaccharides have garnered considerable attention in Chinese medicine research due to their widespread presence and potential therapeutic effects [60–62]. For instance, *Ganoderma lucidum* polysaccharides have demonstrated promising anti-tumor activity, while *Poria cocos* polysaccharides have immune-enhancing effects [63, 64]. Additionally, ginseng polysaccharides, lentinan, fucoidan, pachman, and Coriolus versicolor polysaccharides are already being used as polysaccharide drugs in both domestic and foreign markets [65]. Polysaccharides are complex carbohydrates composed of more than 10 monosaccharides [66]. They are abundantly found in the cell walls of botanical Chinese medicine and the cell membrane of animal Chinese medicine. The biological activities of polysaccharides are closely related to their physicochemical properties, such as monosaccharide composition, glycosidic linkage features, molecular weights, and chain conformations. The solubility of polysaccharides directly affects their hydrolysis, absorption, and subsequent biological effects. Generally, polysaccharides are believed to have low oral bioavailability due to their high molecular weight and low stability in the gastrointestinal tract, leading to their direct excretion through urine in the body [67, 68]. There are three potential ways for the absorption of oral polysaccharides: direct absorption [69–71], transformation by intestinal microflora [72, 73], and absorption by the intestinal Peyer’s aggregated lymph node [74]. Although progress has been made in understanding the in vivo behavior of polysaccharides, the exact mode of oral absorption is still a subject of debate. However, evidence increasingly suggests that transformation by intestinal microflora plays a significant role. In most cases, polysaccharides are absorbed in the form of oligosaccharides. Upon oral ingestion, polysaccharides are hydrolyzed by the intestinal microflora into various monosaccharides and oligosaccharides. These breakdown products are then transported across the intestinal epithelium as monosaccharides or oligosaccharides and subsequently absorbed.

In addition to hydrolysis by intestinal microflora, polysaccharides can also undergo hydrolysis during processing under the influence of heat. This hydrolysis leads to the production of polysaccharides with slightly lower molecular weight, oligosaccharides, and monosaccharides [75]. Polygoni Multiflori Radix and its processed products have been widely used as herbal preparations for medicinal and health products in China and many other East Asian countries for centuries. Polysaccharides are the main components in Polygoni Multiflori Radix, with the weight of the raw product (660.7 kDa) significantly higher than that of the processed product (344.4 kDa) [76]. Honey-processed Astragali Radix (Huangqi in Chinese), which is Astragali Radix processed with honey, exhibits enhanced efficacy in tonifying Qi compared to raw Astragali Radix. Polysaccharides are the main water-soluble active components in honey-processed Astragali Radix. After processing, Astragali Radix polysaccharide (APS3a) undergoes a hydrolysis reaction, converting the 1,4-β-Galpa and 1,6-α-GlcpA aldehyde acid residues into the corresponding neutral residue in honey-processed Astragali Radix polysaccharide (HAPS3a), resulting in a change in molecular weight from 3373.2 to 2463.5 kDa (Fig. 2G) [77]. However, the oral absorption characteristics of processed HAPS3a, such as whether it is absorbed in its prototype or degraded form, and the transport mode involved in its absorption process, have not been extensively researched and remain unclear. Further studies are needed to address these questions.

The effect of heat during steaming on carbohydrate components can be illustrated and explained using oligosaccharides as an example. Rehmanniae Radix is rich in oligosaccharides, including stachyose and raffinose, with stachyose being the most abundant. Stachyose, an oligosaccharide, is rapidly absorbed but less effectively through oral administration, with a bioavailability of less than 4%. Rehmanniae Radix contains a significant amount of stachyose [78, 79]. However, under the influence of heat during processing, the content of stachyose gradually decreases, while the content of fructose, glucose, galactose, and mannose significantly increases [80–82]. One molecule of stachyose can be hydrolyzed into two molecules of galactose, one molecule of fructose, and one molecule of glucose (Fig. 2H) [83]. The hydrolysis of oligosaccharides into monosaccharides during processing (steaming) also contributes to the formation mechanism of Rehmanniae Radix Praeparata, which is described as having a ‘sweet as jelly’ taste.

### Maillard reaction improves protein solubility

The Maillard reaction, also known as glycosylation, is a condensation reaction that occurs between amino-containing components (such as proteins, peptides, or amino acids) and carbonyl-containing components (primarily reducing sugars). Chinese medicine contains significant amounts of proteins, polysaccharides, unsaturated fatty acids, and other components, which makes them prone to undergoing the Maillard reaction during processing. This reaction leads to the formation of Maillard reaction products, which can encapsulate active components and form covalent complexes. The formation of these complexes can offer various benefits, such as improved solubility, emulsification, and stability of the active components. Ultimately, the covalent complexes formed through the Maillard reaction contribute to enhancing the overall quality and efficacy of Chinese medicine.

The Maillard reaction is a complex process that occurs in three stages. In the first stage, a condensation reaction takes place between the free amino group in amino acids or proteins and the carbonyl group in reducing sugars, resulting in the formation of a Schiff base. The Schiff base then undergoes cyclization, leading to the production of N-substituted glucosamine. Through Amadori rearrangement, N-substituted glucosamine transforms into 1-amino-1-deoxy-2-ketose [84, 85]. One notable characteristic of this stage is the conversion of aldose to ketose derivatives. The second stage of the Maillard reaction is still not fully understood, and the principles behind polymer generation in this stage remain unclear. However, certain small-molecule components are known to be produced, including furan rings, nitrogen-containing heterocyclic components, and γ-pyrones.

Recent research has highlighted the pronounced occurrence of the Maillard reaction during the processing (steaming) of red Ginseng Radix et Rhizoma, Polygoni Multiflori Radix (Heshouwu in Chinese) and Rehmanniae Radix [86]. Specifically, during the processing (steaming) of Rehmanniae Radix, there is a rapid decrease in the content of amino acids, especially lysine and arginine. This leads to the formation of various components, including Maltol, 2-ethtylpyrrole, 2,3-dihydro-3,5-dihydroxy-6-methyl-4*H*-pyran-4-one (DDMP), and 5-HMF [87]. One noteworthy finding is that the Maillard reaction can enhance protein solubility. During this reaction, the free amino groups in amino acid residues, particularly lysine and arginine, react with the reducing carbonyl groups of sugar, resulting in covalent cross-linking. This introduces hydrophilic groups, primarily hydroxyl groups, from the sugar, which reduces the isoelectric point of the glycosylated proteins and improves their solubility. The difference in effectiveness between Rehmanniae Radix and Rehmanniae Radix Praeparata may be attributed to the occurrence of the Maillard reaction during processing [88].

## Adjuvants promote the dissolution, absorption, and targeted distribution of active components

To optimize the efficacy of herbs, various liquid adjuvants such as yellow rice wine, vinegar, and honey are commonly employed during processing. Currently, the liquid adjuvants used in processing primarily include wine, vinegar, honey, salt solution, ginger juice, licorice juice, and others.

These adjuvants not only possess therapeutic properties but also enhance the therapeutic effect through compatibility [89]. For example, honey, with its antitussive effects, can synergistically enhance the cough-relieving properties of Ephedrae Herba, leading to prominent effects in relieving cough and asthma [90]. Another common processing method is ginger juice, which enhances the anti-vomiting effect of Bamboo Shavings [91, 92]. In addition to their synergistic effects, adjuvants also play a crucial role in promoting the dissolution, absorption, and targeted distribution of active components in Chinese medicine. These adjuvants help enhance the bioavailability and therapeutic effects of the active components by improving solubility and facilitating absorption in the body.

### Adjuvants promote the dissolution of active components

Wine, vinegar, and honey are commonly employed as solvents to enhance the solubility of various active components in Chinese medicine. When heated, adjuvants such as vinegar, wine, and honey help active components dissolve more easily from complex textures, resulting in improved efficacy. The solubility of active components is influenced by multiple factors, including their structure, solvents, co-existing components, and environmental conditions. Adjuvants can exert various mechanisms to enhance solubility. They can form complexes with the active components, altering their chemical properties and improving their solubility in the chosen solvent. Adjuvants may also act as solubilizers by disrupting the intermolecular interactions within the components, allowing them to dissolve more readily. Overall, the use of adjuvants significantly enhances the dissolution of active components, ensuring their availability and efficacy in Chinese medicine.

### Vinegar increases the dissolution of active components

Processing with vinegar is a traditional technique that effectively enhances the dissolution of active components by altering the solvent. Corydalis is renowned in Chinese medicine for its ability to promote blood circulation and relieve pain. Processing Corydalis Rhizoma (Yanhusuo in Chinese) with vinegar has been shown to significantly enhance its effects of invigorating the circulation of blood, tonifying *Qi*, and relieving pain, as evidenced by the clinical practice of Chinese medicine.

**Fig. 3:**
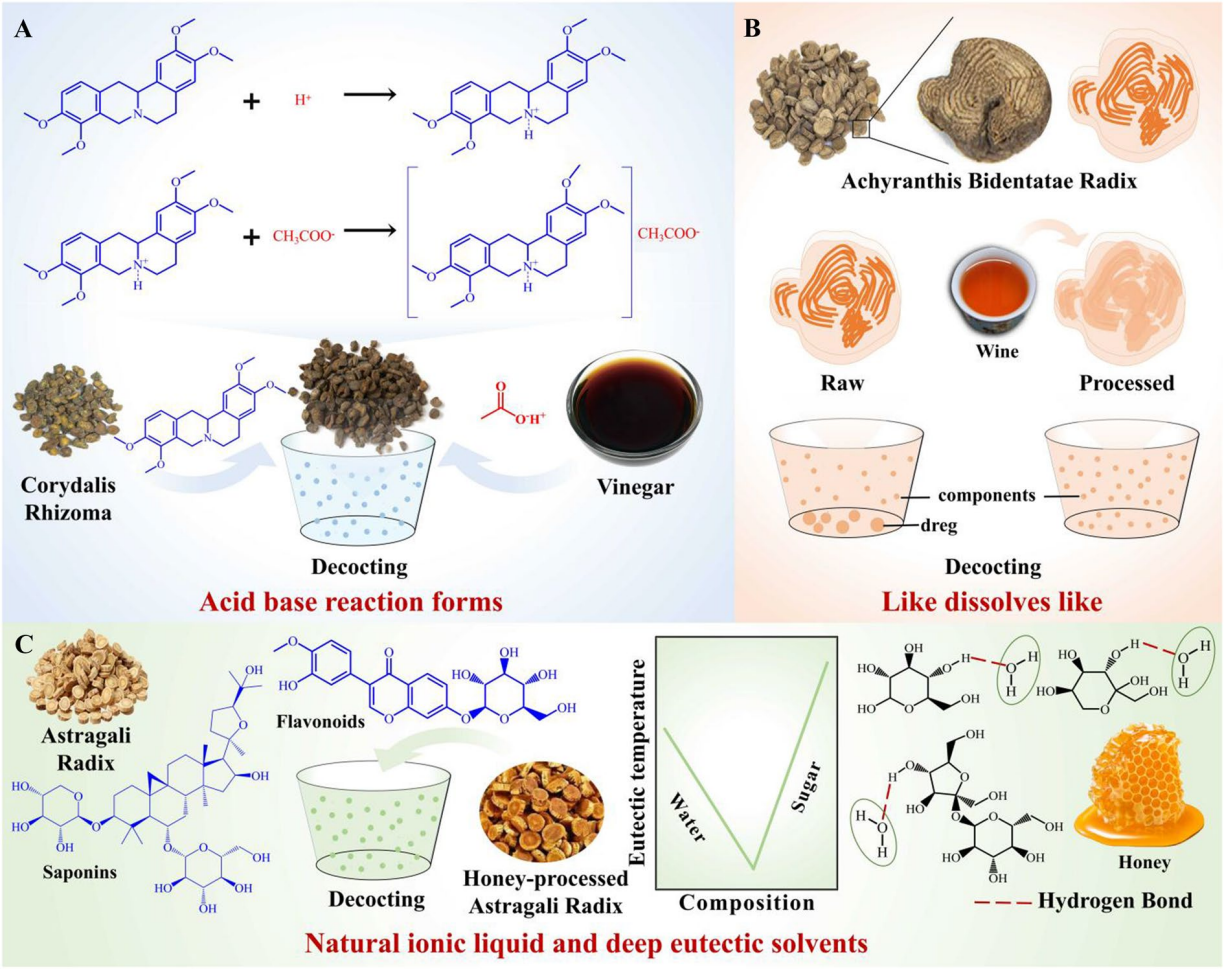
Adjuvants (vinegar, wine, honey) promotes the dissolution of active components. **A** Acetic acid interacts with alkaloids to form acetate salts, increasing their solubility in water; **B** wine processing increases the content of active components in Achyranthis Bidentatae Radix; **C** honey processing increases the water-soluble extract of Astragali Radix

Recent research has revealed the mechanisms by which component solubility is enhanced through processing with vinegar. Acetic acid, a key component of vinegar, interacts with the alkaloid present in Corydalis Rhizoma, forming acetates and thereby increasing the solubility of these components in water [93, 94] (Fig. 3A). Furthermore, processing with vinegar has been found to induce changes in the physical properties of certain active components. For instance, in the case of Olibanum (Ruxiang in Chinese), processing with vinegar alters the surface morphology, reduces particle size and polydispersity index, and decreases viscosity. These alterations contribute to an increased dissolution rate of boswellic acid [95]. Vinegar quenching, a common processing method for mineral and crustacean drugs, is known to modify their crystal form and facilitate decoction. This process also generates acetate, an electrolyte with high solubility. For example, Pyritum (Zirantong in Chinese), which is used for its functions of removing blood stasis, relieving pain, and connecting bones and tendons, contains FeS_2_. Through calcination and vinegar quenching, Pyritum is converted into Fe_2_O_3_ and also produces ferrous acetate, thereby enhancing the solubility of the drugs in decoction [96]. This process also forms ferrous acetate, which increases the solubility of drugs in decoction. These findings highlight the scientific basis behind processing with vinegar, emphasizing its role in enhancing the solubility of active components and improving their therapeutic effects.

### Wine affects the dissolution of active components

Wine is commonly used as an adjuvant in the CMP. Wine processing involves infusing a certain amount of wine into Chinese medicine by various methods, including stir-frying, stewing, steaming, tempering, and quenching. Ethanol, the main component of wine, is a semi-polar organic solvent that dissolves many active components like flavonoids, phenylpropanoids, terpenes, and steroids, which are easily soluble in ethanol. Additionally, ethanol can also dissolve volatile oils, resins, gums, and other insoluble components [97]. As a result, wine has a better extraction effect on low polar components and can enhance the solubility of active components, thereby increasing their extraction and leaching from Chinese medicine.

Achyranthis Bidentatae Radix (Niuxi in Chinese) is a well-known Chinese medicine that is recognized for its various medicinal properties, including expelling blood stasis, clearing the meridians, tonifying the liver and kidneys, strengthening the tendons and bones, inducing diuresis, and draining blood downstream. The wine-processed Achyranthis Bidentatae Radix is a commonly processed product. Researchers have employed chemometrics combined with quantitative analysis using UHPLC-MS/MS to analyze the components of the wine-processed extract. The results showed that wine processing significantly increases the content of certain components in Achyranthis Bidentatae Radix, such as *β*-ecdysterone, 25-R inokosterone, 25-S inokosterone, ginsenoside R0, and chikusetsusaponin IVa, compared to raw Achyranthis Bidentatae Radix [98] (Fig. 3B). For instance, wine treatment has been shown to increase the levels of 15 out of 18 inorganic elements in Corni Fructus (Shanzhuyu in Chinese) [99]. These findings suggest that wine processing can improve the extraction and dissolution of certain insoluble components.

Gentianae Radix et Rhizoma (Longdan in Chinese) is traditionally used in Chinese medicine for clearing *heat* and drying *dampness*, as well as relieving fire in the liver and gall bladder. Studies have demonstrated that the content of active components, such as gentiakochianoside, 1-*O*-glucosyl corymbiferin, macrophylloside A, and oleanolic acid, significantly increases in Gentianae Radix et Rhizoma after being stir-fried with wine. These findings suggest that wine processing can improve the extraction and dissolution of certain insoluble components.

### Honey promotes the dissolution and absorption of active components

Honey is widely used in CMP to enhance therapeutic effects, particularly in terms of *Qi-nourishing* and lung moistening [100, 101]. It primarily consists of saccharides and water, with saccharides making up around 60–80% of its composition. The main saccharides in honey are glucose and fructose, with a small amount of sucrose. These saccharides play a significant role in honey’s physical and chemical properties [102].

Studies have shown that honey forms a supramolecular structure due to the strong hydrogen bond between saccharides and water, giving it similar characteristics to natural ionic liquids and deep eutectic solvents (NADES) [103, 104]. NADES are composed of hydrogen bond donors and acceptors with low vapor pressure [103]. They offer several advantages, including strong solubility, low volatility, and the ability to maintain the stability of solute molecules. Recent research has utilized NADES to improve the solubility and stability of active components (such as saponins, flavonoids, polysaccharides, quinones, alkaloids, phenolic acids, volatile oils, etc.) [105–107]. Both domestic and international studies have confirmed that NADES significantly improve the solubility and extraction rate of phenols, flavonoids, and other components, as well as promote their absorption [108–111].

Astragali Radix is traditionally used for tonifying *Qi* and raising *Yang*, consolidating the surface and stopping sweating, inducing diuresis, reducing swelling, and nourishing blood. Recent studies have shown that Astragali Radix possesses various pharmacological activities, including immunomodulatory, anti-inflammatory, antioxidant, and antitumor effects [112, 113]. In clinical practice, honey-processed Astragali Radix is commonly used as a *Qi-tonifying* and immunomodulating Chinese medicine, exhibiting enhanced tonic effects compared to raw Astragali Radix [114, 115]. Researchers have investigated the mechanism underlying the strengthened tonic effect of honey-processed Astragali Radix, and have reported that honey, along with the influence of heat on chemical components during processing, plays a significant role. Honey processing helps maintain the stability of astragaloside II, increases the water-soluble extract of Astragali Radix, and significantly enhances the content of isoflavone glycosides (e.g., calycosin-7-*O*-*β*-d-glucoside and ononin, etc.) in Astragali Radix [104] (Fig. 3C). These findings explain the superior Qi-nourishing effect of honey-processed Astragali Radix.

### Phase transition induced by adjuvants to increase absorption

Adjuvants can physically change the existing form of active components, which may improve their absorption, and consequently affect their pharmacological effects. This alteration is achieved through non-covalent bonding interactions, including van der Waals forces, hydrogen bonds, π–π superposition, halogen bonds, cation–π interactions, ionic bonds, and CH–π interactions. These interactions facilitate the formation of supramolecular complexes via self-assembly. The formation of such complexes can significantly impact the solubility of insoluble components [116]. Chinese medicine is known for its diverse range of complex components, including flavonoids, saponins, alkaloids, amino acids, and polysaccharides. These components can form aggregates with adjuvants via non-covalent bonding interactions, resulting in the creation of various forms like gels, micelles, spiral bands, and vesicles. These forms serve as solubilizers or permeation enhancers [117].

### The self-assembly of nano micelle of mutton tallow

Mutton tallow, obtained from goats or sheep, is a known adjuvant with tonifying and wind-dispelling properties. Epimedh Folium, a Chinese medicine used for osteoporosis, cardiovascular issues, and impotence, has a long history of use. When processed together with mutton tallow, it can enhance its tonifying deficiency and *Yang* [118–120]. The main active components of Epimedh Folium are flavonoids, which have poor solubility and low bioavailability, thereby impacting clinical efficacy [121–123]. However, by processing Epimedh Folium with mutton tallow, the solubility of icariin can be increased, improving its intestinal absorption and addressing the issue of poor absorption of active flavonoids [124, 125].

Mutton tallow is known to contain fatty acids such as stearic acid, palmitic acids, and oleic acids, which have long fatty chains and exhibit surface activity. Research has found that these fatty acids in mutton tallow can form mixed micelles with sodium taurocholate, an endogenous bile acid. These micelles act as carriers to enhance the absorption of flavonoids [124]. In the case of Baohuoside I, a representative component of Epimedh Folium, it has been observed that the addition of mutton tallow to Baohuoside I-bile salt micelles results in a more stable system. Baohuoside I and bile were able to form micellar particles with an average particle size of (911.2 ± 15.0) nm and zeta potential of (− 11.7 ± 0.5) mV. However, these particles had large polydispersity coefficients and an uneven distribution of particle sizes. The addition of mutton tallow resulted in a significant decrease in the micellar particle size, a more even distribution of particle sizes compared to the Baohuoside I-bile salt micelles, and a significant increase in the zeta potential [126].

**Fig. 4:**
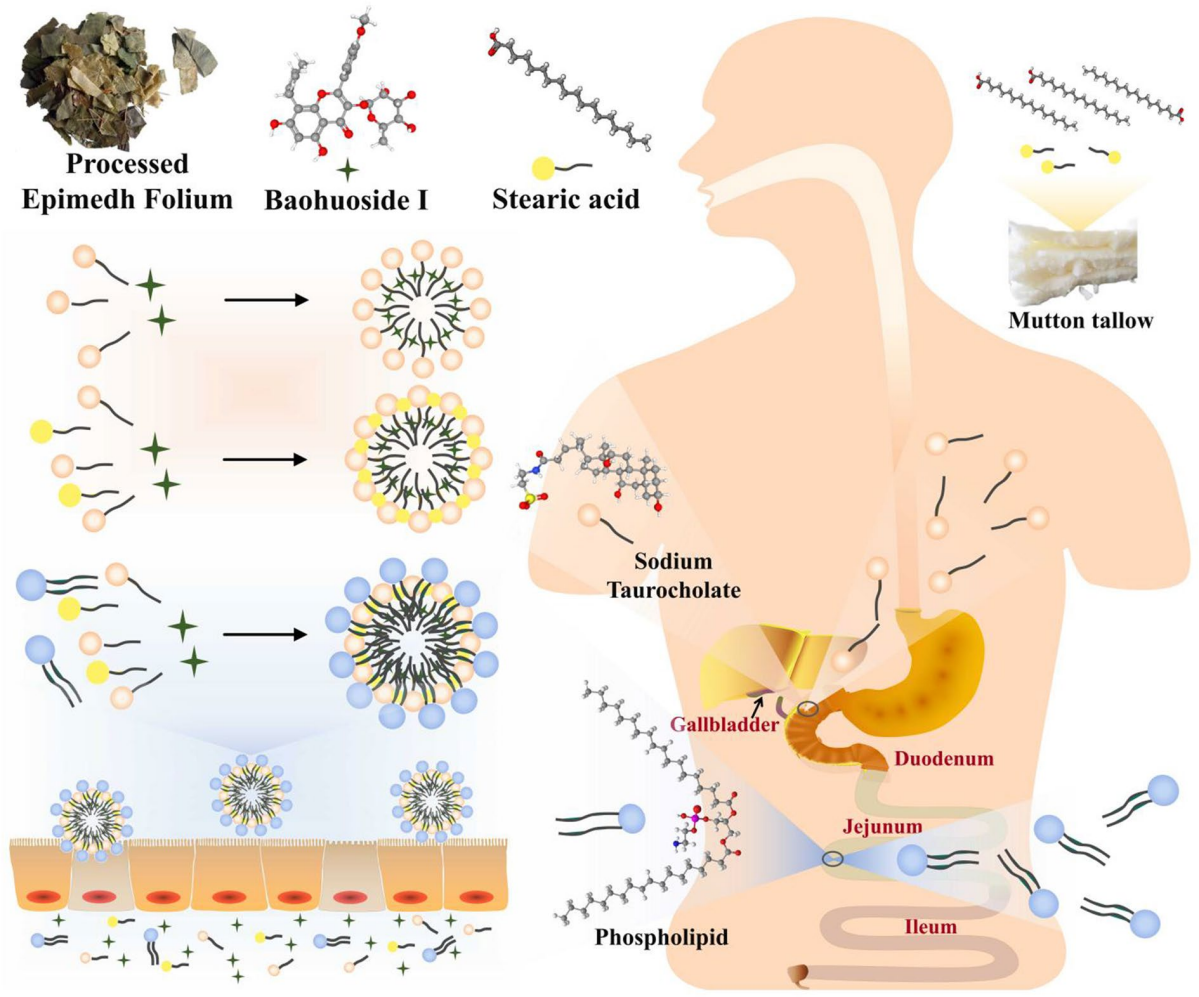
The self-assembly of nano micelle after oral administration of mutton tallow-processed Epimedh Folium

After oral administration of mutton tallow-processed Epimedh Folium, the insoluble active flavonoids can self-assemble with endogenous sodium deoxycholate to form nano micelles. The presence of mutton tallow, which contains stearic acid, palmitic acid, and oleic acid, further promotes the formation of self-assembled micelles, resulting in more stable nano micelles. These nano micelles can then further self-assemble with the action of amphiphilic phospholipids in the body, forming phospholipid-encapsulated nano micelles that play a crucial role in intestinal absorption. This process increases permeability and enhances bioavailability, thereby boosting their synergistic effects. The dynamic self-assembly process significantly improves the solubility and enhances the intestinal absorption of active flavonoids, such as icariin and baohuoside I, ultimately improving their therapeutic efficacy (Fig. 4) [124].

### The self-assembly of nano micelle of bile

Bile, a secretion produced by the liver cells of vertebrates, is commonly used in CMP. Fresh bile derived from cows, pigs, and sheep is known for its heat-clearing, restlessness-relieving, gallbladder-benefiting, detoxifying, liver-clearing, and vision-improving properties. Bile is rich in bile acids and bile salts, which are the main components of bile. These components possess a unique structure with a concave hydrophilic face and a convex hydrophobic face [127]. This structural characteristic allows them to form self-assembled supramolecular nano micelles or micelles [128–130]. Researchers have demonstrated that bile acid or bile salts can self-assemble with lipids or surfactants to form mixed micelles, which exhibit different solubilization capabilities. These mixed micelles act as carriers, enhancing the absorption rate of insoluble components [131, 132].

Coptidis Rhizoma (Huanglian in Chinese) is renowned for its heat-clearing, dampness-drying, fire-dipping, and toxin-removing properties [133]. The main active components of this herb are protoberberine-type alkaloids, including palmatine, coptisine, and jatrorrhizine [134]. Bile-processed Coptidis Rhizoma is a commonly used processed form of this herb, but the mechanism behind its processing with bile has not yet reached a clear consensus. Pharmacokinetic studies have shown that the maximum plasma concentration (C_max_) of berberine and palmatine doubles in bile-processed Coptidis Rhizoma compared to regular Coptidis Rhizoma, suggesting that bile processing enhances alkaloid absorption [135, 136]. Although the mechanism by which bile improves alkaloid absorption in Coptidis Rhizoma requires further investigation, our research team believes that the self-assembly characteristics of bile acids or bile salts may play a key role. The self-assembly properties of bile acids or bile salts, which can form mixed micelles, may contribute to the enhanced absorption of alkaloids in bile-processed Coptidis Rhizoma. However, further research is necessary to fully comprehend the underlying mechanism and elucidate the specific role of bile in improving the absorption of alkaloids from Coptidis Rhizoma.

**Fig. 5:**
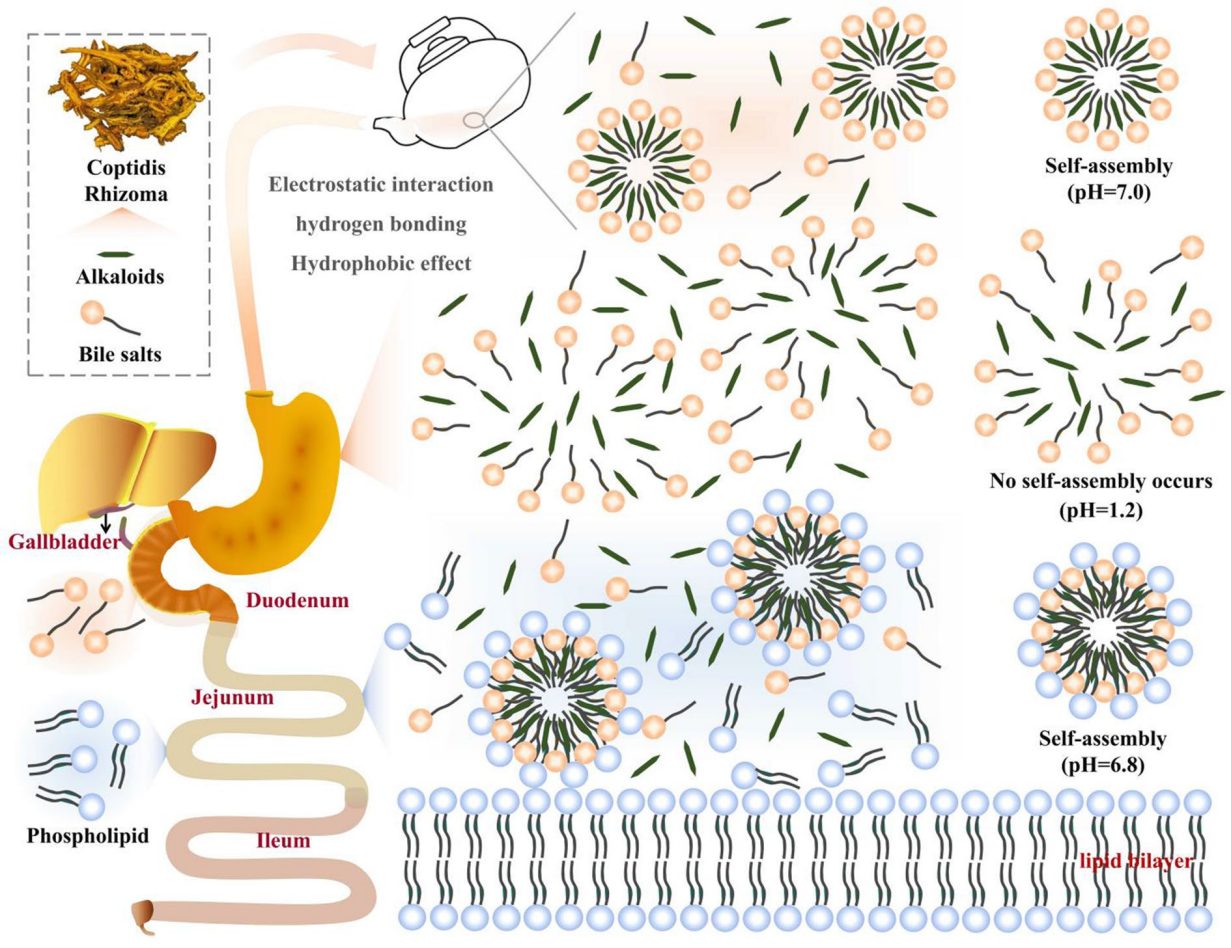
The self-assembly of nano micelle after oral administration of mutton bile-processed Coptidis Rhizoma

Several studies have demonstrated the capacity of bile acids and bile salts to dissolve substances that are typically insoluble. Moreover, phospholipids, which are commonly present in cell membranes, can affect the solubility and stability of these insoluble substances in the intestinal tract. Phospholipids, being amphiphilic, can interact with surfactants like bile salts, resulting in the formation of micelles and lipid aggregates. These structures effectively increase the apparent solubility of insoluble components [137]. The concentration of phospholipids and bile salts in the small intestine has a significant role in shaping the intestinal environment. Research has shown that phospholipids affect the phase behavior of bile components after they are secreted into the duodenum. It has been observed that when the mass fraction of phospholipids is low, the particles formed have a diameter ranging from 3 to 12 nm, but this diameter gradually increases as the amount of phospholipids increases [138]. Therefore, it is believed that the self-assembly properties of bile’s main components (bile salts, bile acids) and the resulting dissolution and absorption of insoluble components, induced by their interaction with phospholipids through phase transition behavior in the intestinal lumen, may contribute to the enhanced potency of the concoction with bile as an adjuvant (Fig. 5). In summary, from a biopharmaceutical perspective, the analysis of the in vivo absorption behavior of insoluble components under bile intervention offers a novel perspective to uncover the scientific basis of CMP.

### The self-assembly of nano micelle of licorice juice

Herbal juices, such as licorice juice, ginger juice, and black bean juice, are commonly used as adjuvants in CMP. Glycyrrhizae Radix et Rhizoma, one of the oldest and most commonly used Chinese medicines, is documented in the pharmacopeias of China, Japan, the US, and Europe. It plays a crucial role in harmonizing and modifying other herbs in a prescription. Licorice juice is an extract obtained through the decoction of Glycyrrhizae Radix et Rhizoma, and its main components include glycyrrhiza polysaccharide, triterpene saponins, glycyrrhiza protein, flavonoids, etc. [139–143]. .

**Fig. 6:**
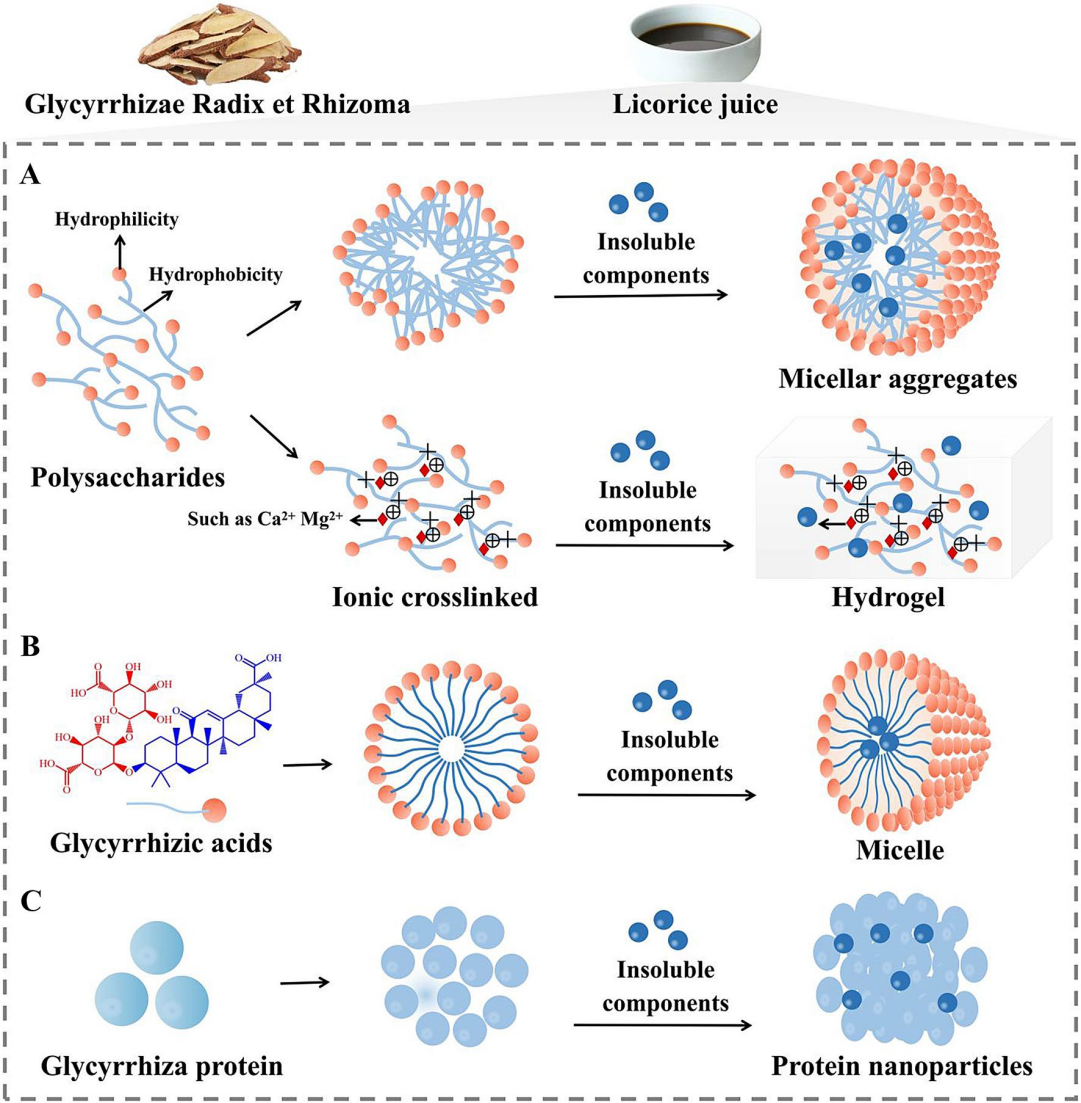
The self-assembly of nano micelle polysaccharides, glycyrrhizic acid and protein in licorice juice. **A** Glycyrrhiza polysaccharides form micellar aggregates and polysaccharide-based hydrogels; **B** glycyrrhizic acids form nano-micelles; **C** glycyrrhiza protein form protein nanoparticles

Glycyrrhiza polysaccharides are key bioactive components found in licorice juice. In recent decades, there has been a growing interest in studying the extraction, separation, and structural characterization of these polysaccharides. The molecular characteristics of polysaccharides play a significant role in their functionality and behavior. They can form nanoparticles with hydrophobic cores and hydrophilic shells in specific solvents, driven by various internal forces such as hydrophobic interactions, van der Waals forces, hydrogen bonds, and electrostatic forces [144]. Glycyrrhiza polysaccharides are known to have complex branched structures and triple helical conformations [145, 146]. These structures can form complex arrangements and encapsulate active small molecules, resulting in the formation of spherical aggregates [147–149]. Moreover, glycyrrhiza polysaccharides can interact with metal ions through ion interactions, leading to the formation of hydrogels with three-dimensional networks and hydrophilic porous structures (Fig. 6A) [150, 151]. Previous studies have demonstrated that glycyrrhiza polysaccharides have the potential to enhance the stability and solubility of aconitine, hypaconitine, and benzoylmesaconine in vitro. Furthermore, glycyrrhiza polysaccharides have been found to improve the bioavailability and increase the elimination rate of aconitine, thereby allowing for a reduction in the clinical dosage and mitigating its toxicity. Additionally, glycyrrhiza polysaccharides have been observed to prolong the presence of hypaconitine in the body, thereby extending its therapeutic effect, and enhancing the bioavailability of benzoylmesaconine [152].

Moreover, licorice juice also contains triterpene saponins, which are amphiphilic components consisting of non-polar saponins and water-soluble side chains. During the decoction process, these triterpene saponins can form nanoparticles, thereby increasing the solubility of insoluble components [153]. Glycyrrhizic acid, a typical triterpene saponin found in licorice juice, consists of a hydrophobic triterpene and a hydrophilic sugar chain containing two glucuronides and one carboxyl group [154]. This amphiphilic nature allows glycyrrhizic acid to self-assemble and form non-covalent complexes through hydrophobic interaction [155] (Fig. 6B). Dimeric complexes of glycyrrhizic acid can be observed in solution at concentrations ranging from 0.01 to 1 mmol/L, while at concentrations exceeding 1 mmol/L, large micelle-like aggregates can be formed [156, 157]. At a critical micelle concentration, hydrophobic chain segments (glycoside) form the inner core, while hydrophilic chain segments (sugar chains) form the outer shell, driven by a non-covalent bonding force. This self-assembled micelle effectively enhances the solubility of insoluble components. For instance, the glycyrrhizic acid dimer, formed through the self-assembly of glycyrrhizic acid, can bind hydrophobic molecules on its ring surface, creating a “host-guest complex” that enhances the solubility of hydrophobic components [158]. Studies have demonstrated that glycyrrhizic acid can form a ‘micellar phase’ with puerarin, berberine, and baicalin in Ge-Gen-Qin-Lian-Tang decoction, increasing solubility and absorption [159]. Current research on the solubilization mechanism of glycyrrhizic acid primarily focuses on its supramolecular self-assembly properties, which may explain its solubilization mechanism for certain hydrophobic active components.

Research on the chemical and structural characteristics of nanoparticles indicates that macromolecular components, such as proteins and polysaccharides, often form the primary framework [160, 161]. Proteins, which play a fundamental role in living organisms, exert their biological functions mainly through supramolecular self-assembly [162]. Extensive studies have been conducted on glycyrrhiza proteins, which are rich in tyrosine and tryptophan, revealing their self-assembly behavior during the decoction process to form spherical nanoparticles [163]. It is important to note that proteins achieve their effects through self-assembly via non-covalent interactions. Amino acid residues, acting as weak electrolytes, are distributed on the protein surface, resulting in charge variations at different pH values. This property enables the attraction of electrostatic interactions and facilitates large-scale protein self-assembly [164]. Additionally, glycyrrhiza protein has been observed to encapsulate insoluble components through weak bonding, such as hydrophobic interactions and electrostatic interactions between amide groups and quaternary ammonium ions. This leads to the formation of a subspherical shape [165] (Fig. 6C). The berberine-licorice protein conjugate exhibits a significantly different microscopic morphology compared to the two monomers. It presents a clear globular-like structure with an average particle size of approximately 185.5 nm and a polydispersity index of 0.285, indicating a large-scale self-assembly behavior [165]. The existing body of evidence suggests that glycyrrhiza polysaccharide, triterpene saponins, and glycyrrhiza protein found in licorice juice have the potential to enhance the solubility and absorption of active components. This is achieved by forming aggregates, hydrogel, or micelles via self-assembly. Consequently, their self-assembly characteristics and their ability to improve the solubility and permeability of active components may play a crucial role in their utilization as adjuvants in processing.

### Adjuvants promote the targeted distribution of active components

During the processing of Chinese medicine, adjuvants are carefully chosen based on their specific properties and effects. Wine is used to promote upward movement and cleanse upper-energizer heat, vinegar enhances liver-soothing and analgesic effects, honey increases Qi-nourishing and lung-moistening effects, and salt solution facilitates the delivery of drugs to the kidneys. The concept of meridian tropism in adjuvant processing shares similarities with targeted preparations in pharmacology, as both aim to modify drug behavior in the body to increase their effectiveness. In recent years, extensive research has focused on the scientific implications of the high-concentration distribution of active components in target organs and their potentiation under the influence of adjuvants.

### Salt solution promotes active component distribution in the kidney

Salt mainly contains NaCl and trace amounts of MgCl_2_, CaCl_2_, KCl, NaI, and other components. Recent research has shed light on the vital physiological roles of sodium ions (Na^+^) in salt. These ions are essential for maintaining osmotic pressure, facilitating nerve and muscle cell excitation, and aiding nutrient absorption in the gastrointestinal tract [166]. Highly concentrated salt solution is commonly used in processing [167]. Salt solution-frying and salt solution-steaming are the primary techniques mentioned in the Chinese Pharmacopeia (2020 edition) and regional processing norms [168]. According to Chinese medicine theory, processing with salt solution can enhance the transportation of active components to the kidney meridian, thereby enhancing their therapeutic effects on lower jiao disorders [169, 170].

Psoraleae Fructus (Buguzhi in Chinese) is well-known for its ability to ‘warm and invigorate the Kidney-Yang, gather the spirit, and enrich the bone marrow’, as described in the Compendium of Materia Medica. In clinical practice, salt solution-processed Psoraleae Fructus is commonly used in Chinese medicine, and has been shown to enhance its kidney-warming and yang-tonifying effects. This enhancement may be attributed to the effects of salt solution-frying on the absorption behavior of the major components [171–173]. Among these components, psoralen and isopsoralen are the two isomers and main active components within Psoraleae Fructus [174]. Processing with the salt solution significantly increases the area under the curve (AUC) for psoralen and isopsoralen and promotes their distribution in the kidneys and related reproductive organs [175, 176]. The use of salt treatment has been shown to enhance the uptake of psoralen and isopsoralen, leading to their increased distribution in various organs such as the heart, liver, spleen, lungs, kidneys, ovaries, and testes, with the reproductive organs experiencing the greatest increase. A comparison of the AUC value revealed that the distribution of isopsoralen in the ovary and testis, as well as the distribution of psoralen in the ovary more than doubled [175].

Morindae Officinalis Radix (Bajitian in Chinese) is renowned for its ability to tonify kidney yang, alleviate rheumatism, and strengthen muscles and bones. Raw Morindae Officinalis Radix is known to dispel wind and dampness, while salt solution-processed Morindae Officinalis Radix enhances its kidney-tonifying and Yang-strengthening effects. The main active components of Morindae Officinalis Radix are Iridoid glycosides, with monotropein being the most abundant [177, 178]. Researchers have investigated the impact of salt solution stir-frying on the distribution of monotropein in Morindae Officinalis Radix and have found that salt solution processing can improve the distribution of monotropein and promote its presence in kidney tissue. These findings align with the traditional theory of ‘salt solution-processing entering the kidney meridian’ [179].

Extensive research has revealed that after undergoing salt processing, the active components are distributed specifically in the kidneys and related reproductive organs, resulting in *kidney-tonifying* effects. However, the exact mechanisms behind this phenomenon are still not fully understood. The kidney plays a pivotal role in maintaining fluid and electrolyte balance in the body [180]. The renal reabsorption of Na^+^ is a crucial physiological process carried out by the kidneys [181]. In clinical practice, a significant number of active components in salt-processed medications are absorbed by the kidneys through Na^+^ reabsorption. Therefore, the targeted distribution of active components following salt processing may be attributed to their absorption by the kidneys via Na^+^ reabsorption.

### Wine affects the upward distribution of active components

According to historical records from the Yuan Dynasty, it was mentioned that when a disease affects the head, face, or surface of limb muscle, Chinese medicine should be stir-fried with wine to allow it to ascend via the power of wine. This indicates that wine can alter the nature of Chinese medicine by promoting an upward direction and cleaning upper-energizer heat. Recent studies have shown that wine facilitates the entry of more active components into the bloodstream, thereby promoting an upward direction and enhancing its ascending efficacy. Scutellariae Radix (Huangqin in Chinese) is a widely used Chinese medicine known for its ability to clear heat, and dry dampness, relieve fire, and detoxify the body [182]. Wine-processed Scutellariae Radix is a specific product derived from Scutellariae Radix and is commonly used in clinical practice to clear upper-energizer heat [183]. The main active components of Scutellariae Radix are flavonoids [184, 185]. Pharmacokinetic studies have revealed that wine processing significantly increases the C_max_ and area under the curve (AUC_0−t_) of certain flavonoids in upper-energizer tissues such as the lungs and heart, while significantly decreasing in middle-energizer and lower-energizer tissues such as the spleen, liver, and kidney [186]. The distribution of flavonoids in the tissues is consistent with the ascending and descending theory, indicating that wine has an ‘induce medicine upward’ effect and concentrates active components in the upper-energizer tissues.

## The modern research system for the synergistic mechanism of CMP

**Fig. 7:**
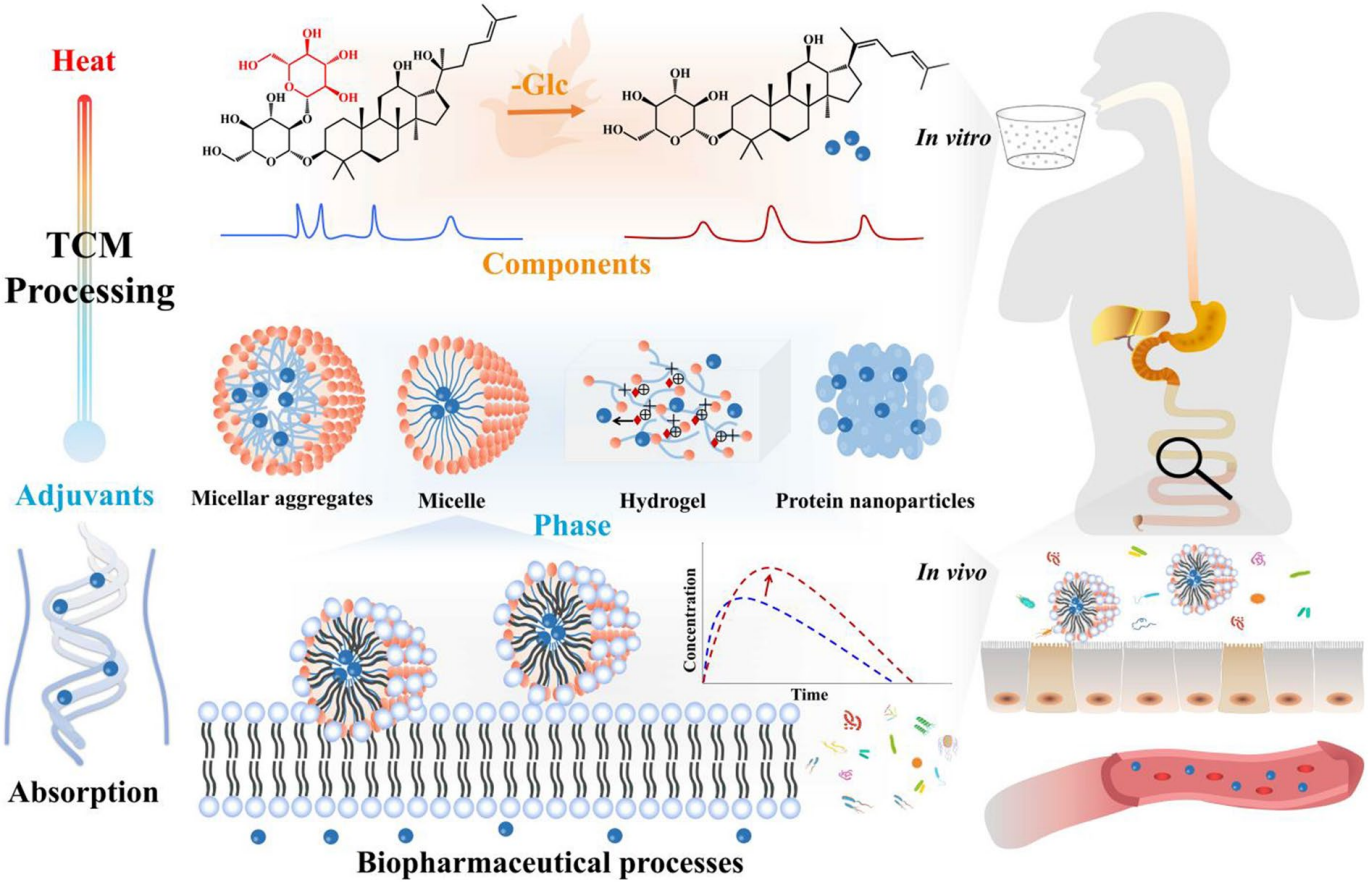
The modern research system for the synergistic mechanism of CMP

The paper proposes a modern research system to understand the synergistic mechanism of CMP, which encompasses three key aspects: (1) the changes in chemical components that occur during processing; (2) the formation of different phase transitions (micelles, aggregates, etc.) under the intervention of the components themselves or adjuvants used in the preparation process, like decoction; (3) the absorption, distribution and metabolic characteristics of active components in vivo, influenced by the adjuvants in the biopharmaceutical process (Fig. 7).

Previous research on the mechanism of CMP has predominantly focused on changes in the ‘content’ of components caused by heat and adjuvants during processing. The increase in the concentration of active components has been considered an important factor contributing to the synergistic effect of CMP. However, recent studies have highlighted that the conversion of chemical components during processing can result in alterations in biopharmaceutical properties, such as solubility and permeability. The active components that exhibit enhanced absorption (with appropriate solubility and permeability) after processing are closely linked to their synergistic effect. Therefore, investigating the conversion of chemical components during processing and its impact on improving the biopharmaceutical properties of active components represents a significant aspect of research into the synergistic mechanism of CMP.

The preparation process, which serves as an intermediate step between CMP and the clinical administration of Chinese medicine, has often been overlooked in previous research on the mechanism of CMP. However, it is crucial to consider that active components within Chinese medicine can form nano aggregates through non-covalent bonds. For example, sugar components are rich in hydrophilic groups, allowing them to self-assemble with other structural units through hydrophobic or hydrogen bonding [187]. Triterpene components, with their rigid skeleton and multiple chiral centers, can easily fold into different forms, forming self-assembled nanoparticles in various media. Current research has discovered that self-assembly behavior can occur within the same component or between different components of Chinese medicine [188, 189]. During the processing, preparation, and biopharmaceutical processes of Chinese medicine, the phenomenon of multi-component self-assembly is observed. Furthermore, adjuvants also possess certain special components that can form a unique form with the active components, altering their solubility and permeability, and thereby influencing their intestinal absorption behavior. Therefore, studying the formation of different phase transitions, such as micelles and aggregates, under the influence of the components themselves or adjuvants during the preparation process (decoction), and their impact on the intestinal absorption behavior of active components, represents an important aspect of research into the synergistic mechanism of CMP.

Oral administration is the primary method of clinical use for Chinese medicine. The absorption of Chinese medicine through oral administration mainly occurs in the small intestine. The chemical components of Chinese medicine may interact with each other within the intestinal absorption barrier network, thereby before and after preparation, influencing their absorption. Therefore, understanding the biopharmaceutical processes of absorption and transit of orally administered Chinese medicine in the gastrointestinal tract is crucial to comprehend their biological effects. By investigating the changes in chemical components in vitro, analyzing the absorption barrier network of the gastrointestinal tract, and exploring the impact of intestinal flora on the absorption, distribution, and metabolism of chemical components, we can delve into the scientific connotations of the CMP from various aspects. We propose establishing a three-dimensional research system, encompassing ‘chemical reaction—phase transition—biopharmaceutical properties’. This system integrates the changes in chemical components during the CMP process, the existing state of active components during preparation, and the absorption, distribution, and metabolism of components during the biopharmaceutical process. Only through such an approach can we provide a comprehensive and multi-level research system to investigate the mechanism of CMP, comprehensively explain how CMP enhances efficiency, and avoid the paradox where the increase or decrease of components after processing contradicts their efficacy.

### Summary and outlook

The efficacy of Chinese medicine is not only dependent on its active components but also on its physical state and biopharmaceutical properties. Analyzing the physical state and self-assembly behavior of active components during different stages, including processing, boiling, and in vivo absorption, provides a comprehensive understanding of the CMP mechanism. The self-assembly behavior between adjuvants and active components has become a research focus. In most cases, adjuvants can synergize with active components to enhance efficacy and increase solubility. This paper provides a biopharmaceutical perspective on the self-assembly behavior between adjuvants and active components, emphasizing their positive role in component synergy and the enhancement of efficacy by increasing the solubility of insoluble or poorly soluble components.

The absorption of active components is influenced by changes in their physical properties and increased solubility. Biopharmaceutics and pharmacokinetics theories and methods are commonly used to better understand the transmission mechanism between the existing forms of active components and their biopharmaceutical properties in vivo. These theories and methods help investigate how active components are absorbed, distributed, and metabolized in different forms. In recent years, research on the in vivo absorption, distribution, and metabolism of active components has become essential in comprehending the mechanism of CMP. Additionally, there is a growing interest in understanding the processing mechanism from the perspective of transporters, metabolic enzymes, and other in vivo aspects.

In the study of the mechanism of CMP, researchers should adhere to the principle that Chinese medicine originates from clinical practice and serves the clinic, fully considering the clinical needs of Chinese medicine and its application form. In addition to focusing on the active components, researchers should also analyze the impact of the gastrointestinal absorption barrier network and intestinal bacterial flora on the absorption, transportation, metabolism, and changes of the active components within the body. The proposed three levels of “chemical reaction—phase transition—biopharmaceutical properties” in this paper present a comprehensive and integrated research strategy for exploring the mechanism of CMP. The interpretation of the enhanced efficacy of CMP from a biopharmaceutic perspective not only provides a novel viewpoint but also offers a valuable research strategy. This strategy can greatly contribute to guiding the study of CMP mechanisms, quality control of Chinese medicine, quality standardization of decoction pieces, and the development of new medicines in Chinese medicine.

## Data Availability

Not applicable.

## Abbreviations

CMP: Chinese medicine processing
APS3a: Astragali Radix polysaccharide
HAPS3a: Honey-processed Astragali Radix polysaccharide
DDMP: 3,5-dihydroxy-6-methyl-4 *H*-pyran-4-one
NADES: Natural deep eutectic solvents
AUC: The area under the curve
